# The mitochondrial Ahi1/GR participates the regulation on mtDNA copy numbers and brain ATP levels and modulates depressive behaviors in mice

**DOI:** 10.1186/s12964-022-01034-8

**Published:** 2023-01-23

**Authors:** Bin Wang, Haixia Shi, Bo Yang, Zhigang Miao, Miao Sun, Hao Yang, Xingshun Xu

**Affiliations:** 1 Department of Fetology, The First Affiliated Hospital of Soochow University, Suzhou, 215006 China; 2 Institute of Neuroscience, Soochow University, Suzhou, 215123 China; 3 Department of Anesthesiology, The Second Affiliated Hospital of Soochow University, Suzhou, 215004 China; 4 Department of Neurology, The First Affiliated Hospital of Soochow University, Suzhou, 215006 China; 5 Jiangsu Key Laboratory of Neuropsychiatric Diseases, Soochow University, Suzhou, 215123 Jiangsu China

**Keywords:** Ahi1, Glucocorticoid receptor, Mitochondrial transcription factor A, Mitochondrial DNA, ATP, Exercise

## Abstract

**Background:**

Previous studies have shown that depression is often accompanied by an increase in mtDNA copy number and a decrease in ATP levels; however, the exact regulatory mechanisms remain unclear.

**Methods:**

In the present study, Western blot, cell knockdown, immunofluorescence, immunoprecipitation and ChIP-qPCR assays were used to detect changes in the Ahi1/GR-TFAM-mtDNA pathway in the brains of neuronal Abelson helper integration site-1 (Ahi1) KO mice and dexamethasone (Dex)-induced mice to elucidate the pathogenesis of depression. In addition, a rescue experiment was performed to determine the effects of regular exercise on the Ahi1/GR-TFAM-mtDNA-ATP pathway and depression-like behavior in Dex-induced mice and Ahi1 KO mice under stress.

**Results:**

In this study, we found that ATP levels decreased and mitochondrial DNA (mtDNA) copy numbers increased in depression-related brain regions in Dex-induced depressive mice and Ahi1 knockout (KO) mice. In addition, Ahi1 and glucocorticoid receptor (GR), two important proteins related to stress and depressive behaviors, were significantly decreased in the mitochondria under stress. Intriguingly, GR can bind to the D-loop control region of mitochondria and regulate mitochondrial replication and transcription. Importantly, regular exercise significantly increased mitochondrial Ahi1/GR levels and ATP levels and thus improved depression-like behaviors in Dex-induced depressive mice but not in Ahi1 KO mice under stress.

**Conclusions:**

In summary, our findings demonstrated that the mitochondrial Ahi1/GR complex and TFAM coordinately regulate mtDNA copy numbers and brain ATP levels by binding to the D-loop region of mtDNA Regular exercise increases the levels of the mitochondrial Ahi1/GR complex and improves depressive behaviors.

**Video Abstract**

**Supplementary Information:**

The online version contains supplementary material available at 10.1186/s12964-022-01034-8.

## Introduction

Depression is a common psychiatric disorder that severely limits psychosocial functions and reduces quality of life. In 2008, the WHO ranked major depression as the third-leading cause of the global burden of diseases and predicted that it will rank first by 2030 [1, 2]. Since the pathogenesis of depression is not clear, only approximately 60% of patients with major depression respond effectively to currently marketed antidepressant agents [3]. Therefore, exploring the pathogenesis of depression and finding fast, safe, and effective antidepressants are still key scientific issues in current research on depression. An increasing amount of studies have shown that depression is often accompanied by the impairments in mitochondria, such as increased mtDNA copy number and decreased ATP levels [4–6], but the exact mechanisms remain to be elucidated.

The AHI1 gene plays a key role in brain development, and mutations in *AHI1* can lead to Joubert syndrome, a rare autosomal recessive disorder characterized by abnormal brain development and mental retardation [7]. Our previous study found that Ahi1 knockout (KO) mice exhibit severe depression-like behaviors [8, 9], and Ahi1 interacts with glucocorticoid receptor (GR) to affect its translocation from the cytoplasm to the nucleus, thereby altering the GR-mediated stress response [10]. Interestingly, previous studies have also shown that GR enters the mitochondria and binds to the D-loop control region of mtDNA to initiate or suppress mitochondrial gene expression [11–13]. Long-term treatment with high-dose corticosterone in rats leads to a significant decrease in mitochondrial GR levels in the prefrontal cortex [14]. However, whether Ahi1 promotes the translocation of GR into mitochondria and its regulation of mitochondrial gene expression remain to be confirmed.

Similar to GR, mitochondrial transcription factor A (TFAM) protein can also bind to the D-loop control region of mitochondria and regulate mitochondrial replication and transcription [15–18]. TFAM is a nuclear-encoded transcription factor that is synthesized in the cytoplasm and imported into the mitochondria [19]. It is vital for the regulation of mitochondrial gene expression and the maintenance of mitochondrial functions. A previous study showed that heterozygous TFAM knockout in mice reduces mitochondrial DNA (mtDNA) copy number and leads to respiratory chain deficiency, whereas homozygous knockout is embryonically lethal [20]. Conversely, TFAM overexpression elevates mtDNA abundance, indicating the strong correlation between TFAM levels and mtDNA copy number [17, 21]. However, whether TFAM affects GR binding to the D-loop control region of mtDNA to regulate mitochondrial gene expression still needs further investigation.

The function of mitochondria is related to the integrity and quantity of mtDNA. Abnormal mtDNA is associated with a variety of human diseases, including obesity, cardiomyopathy, and cancer [4, 5, 22–25], as well as neurodegenerative and neuropsychiatric disorders [4, 26–28]. Because the biogenesis of the oxidative phosphorylation system requires the adequate expression of mtDNA [29], mtDNA mutation and copy number changes are important indicators of mitochondrial dysfunction [30]. Previous studies demonstrated that the increase in total mtDNA copy number by TFAM overexpression ameliorates the pathological consequences of a heteroplasmic mtDNA mutation in animal models [31, 32]. In contrast, TFAM overexpression causes detrimental effects by impairing mtDNA transcription and results in progressive dysfunction of the oxidative phosphorylation system [30, 32]. Consistent with this, energy production is reduced in stressed mice with elevated mtDNA levels under a depressed state [4]. To date, it remains unclear whether TFAM plays a critical role in the regulation of mtDNA levels in depressive behaviors.

Regular physical exercise improves depressive behaviors in numerous clinical studies [33, 34]; animal studies have also shown that exercise training improves depression-like behaviors [35–38]. However, the biological mechanisms of exercise remain incompletely understood. Previous studies have found that exercise activates mitochondrial pathways and plays an antidepressant role [38, 39]. Under stress or depressive conditions, low extracellular ATP is accompanied by a reduction in total ATP production, while stimulating endogenous ATP release or exogenous ATP administration produces antidepressant-like effects [6, 40–42]. However, it is unclear whether regular exercise and exogenously added extracellular ATP are effective in improving the depressive behaviors of Ahi1 KO mice.

In this study, we found that mitochondrial Ahi1/GR coordinately regulates mtDNA copy numbers and ATP production. In addition, exercise significantly increased mitochondrial Ahi1/GR levels and ATP levels, resulting in the improvement of depression-like behaviors in Dex-induced depressive mice under stress; however, depression-like behaviors and ATP levels were not improved in Ahi1 KO mice under stress.

## Materials and methods

### Animals

Ahi1 knockout (KO) mice were produced as previously described [8]. Ahi1^loxp/loxp^ mice were crossed with mice carrying an EIIa promoter-driven Cre transgene, and the resulting heterozygous mice were used to generate homozygous conditional KO mice. All mice were maintained in cages with a maximum of five mice at 22 ± 2 °C in a 12 h light (7 am–7 pm)/dark cycle (7 pm–7 am) and free access to food and water at Soochow University Experimental Animal Center. All procedures and protocols in this study were performed according to the Animals Act, 2006 (China) and approved by the Institutional Animal Care and Use Committee of Soochow University. Because heterozygous Ahi1 conditional knockout mice (nes-Ahi1^+/−^) carry the Cre transgene and are closer to homozygous Ahi1 conditional knockout mice (nes-Ahi1^−/−^), they had no significant difference in Ahi1 expression and behaviors compared with wild-type mice; therefore, we used nes-Ahi1^+/−^ mice as controls.

### Dex-induced depressive mouse model

The Dex-induced depressive mouse model was previously described [10]. To induce depressive behaviors in mice, 2-month-old male ICR mice (25–30 g) were exposed to subcutaneous injections of Dex (1 mg/kg) or normal saline (NS) for 21 days. Depression-like behavior tests were performed by a trainee who was blinded to the groups of mice through behavioral experiments.

### Exercise training

Ahi1 KO mice and Dex-induced depressed mice were divided into the exercise group and the nonexercise group. Mice in the exercise group underwent 4 weeks of training consisting of 5 running sessions per week (Monday to Friday) of treadmill exercise on a treadmill apparatus as previously described [43, 44]. Each running session lasts 40 min/day, with treadmill speed starting at a protocol of 6 m/min and gradually increasing treadmill speed by 2 m/min/week. Mice in the control (sedentary) group were placed on a locked treadmill for the same period of time.

### Forced swim test (FST)

The forced swim test was performed as previously described [9]. Each mouse was placed in a glass cylinder (20 cm high and 15 cm in diameter) with water (23–25 °C) approximately 14 cm deep. The immobility time was recorded for 6 min. A trainee who was blinded to the genotypes or the groups of the mice manually recorded the immobility time with a stopwatch. Immobility was defined as floating in the water without moving, except for minimum movements necessary to keep the head above water. The water was changed between each trial.

### Tail suspension test (TST)

The tail suspension test was carried out according to previously described methods [45, 46]. The tail of mice was tapped 35 cm over a table by adhesive tape. The mice were suspended for 6 min and the time of immobility was recorded by a trainee who was blinded to the genotype or the group of mice.

### Sucrose preference test (SPT)

Mice were housed individually and trained to drink two bottles of water for 24 h. On the second day, one bottle of water was replaced with a bottle of 1% sucrose solution. After 24 h, the positions of the two bottles were exchanged. On the fourth day, mice were deprived of both food and water for 24 h. The bottles were weighed prior to water and food provision. Twenty-four hours later, the bottles were weighed again. The percentage of sucrose consumed to the total drink was calculated by a trainee who was blinded to the genotype or the group of mice.

### Animal drug administration

Imipramine (IM; Sigma-Aldrich Corp., St Louis, MO, USA) or ATP (Sigma-Aldrich Corp., St Louis, MO, USA) was dissolved in normal saline (NS) and freshly prepared before treatment. Mice were intraperitoneally treated with IM (20 mg/kg), ATP (125 mg/kg), or NS for 3 weeks.

### PC12 cell culture

PC12 cells were maintained in Dulbecco’s modified Eagle’s medium (HyClone, Logan, UT, USA) with 10% FBS (Gibco BRL Co. Ltd., USA), 100 UI/ml penicillin sodium, and 100 μg/ml streptomycin sulfate. When PC12 cells reached 50–60% confluence, the cells were treated with Dex/mifepristone or transfected with Ahi1/GR siRNA for 48 h. The siRNAs were synthesized by Gene Pharma and brought into the PC12 cells by transient transfection with Lipo2000 (Thermo Scientific). After treatment, PC12 cells were collected for Western blotting analysis.

### Isolation of mitochondria

Mitochondria were isolated from mouse brains or PC12 cells according to previously described methods [47, 48]. Fresh brain tissue or cells were homogenized in a 2-ml glass Dounce tissue grinder (Kontes Glass Co., Vineland, NJ, USA) containing 0.95 mL 12 vol% Percoll. The homogenate was carefully added to the 40% and 19% Percoll gradients and centrifuged at 30,700 × g at 4 °C for 7 min to produce a dense band (band 3, mitochondria-enriched fraction) between the two lower Percoll layers. After careful removal of bands 1 and 2, band 3 was collected and diluted with 1 ml IB (10 mM Trizma base, 320 mM sucrose, 1 mM EDTA, pH 7.4) and then centrifuged at 16,700×*g* for 12 min. After removal of the supernatant, the mitochondrial precipitate was carefully transferred to a microfuge tube (Eppendorf) containing 400 μL IB and 100 μL BSA (1 mg/ml) and carefully mixed at 7300×*g* for 6 min at 4 °C, producing dense mitochondrial particles. The final mitochondrial pellet was used for further analysis.

### ATP detection

Fresh brain tissue was dissociated (within 5 min), and PC12 cells were collected within 2 min by a trained person who was blinded to the genotype or the group. The ATP levels were measured with an enhanced ATP assay kit (S0027 Beyotime Biotechnology, Shanghai, China) according to the manufacturer's instructions. In brief, the collected samples were rapidly frozen in liquid nitrogen for 30 s and homogenized in lysis buffer for 1 min on ice. Lysates were collected and centrifuged at 12,000×*g* for 5 min at 4 °C. In a 96-well plate, 20 μl of supernatant was added to the wells containing 100 μl of ATP assay working dilution. Luminescence was detected by a multifunctional microplate reader (Infinite M200 PRO; Tecan, Switzerland). The protein concentration of each group was determined and used to calibrate the ATP levels in the cells. Finally, the ATP concentration was calculated through the derived ATP standard curve and normalized to the protein concentration of the supernatants.

### Agilent seahorse XF real-time ATP rate assay

PC12 cells (2X10^4^ cells/well) were seeded in 24 well Seahorse XF24 Cell Culture Microplates and transfected with Con-siRNA or Ahi1-siRNA for 48 h. Cells were washed with ATP Assay Medium (Agilent XF DMEM Medium pH 7.4; 10 mM XF Glucose; 1 mM XF Sodium Pyruvate; 2 mM XF L-Glutamine) and incubated with warm ATP Assay Medium at 37 °C (non-CO_2_) for 45 min. The medium was removed and replaced with fresh ATP Assay medium. Then, the plates were placed on the Seahorse XFe24 Extracellular Flux Analyzer. The ATP production rate was measured under stress conditions in response to 1.5 μM of oligomycin and 0.5 μM rotenone plus antimycin A according to the manufacturer’s instructions (Agilent 103,592–100). The ATP production rate was normalized to the protein concentrations. Data were analyzed in the Agilent ATP Assay Report Generator, and statistical analyses were performed using GraphPad Prism 8.0.

### Citrate synthase and isocitrate dehydrogenase activity assay

Citrate synthase and isocitrate dehydrogenase activity were determined with a Citrate Synthase Activity Assay Kit (MAK193) and isocitrate dehydrogenase activity assay kit (MAK062) (Sigma, St. Louis, MO, USA) in accordance with the manufacturer’s instructions.

### Determination of cell viability

Cell viability was evaluated by the ability to metabolize 3-(4, 5-dimethylthiazol-2-yl)-5-(3-carboxymethoxyphenyl)-2(4-sulfophenyl)2-H-tetrazolium, inner salt (MTS) following the standard procedure in our previous experiments [49]. Cells were inoculated onto 96-well plates, followed by Dex (20 μM) treatment for 72 h. Concomitantly 10 μl of MTS solution was added to each well and the cells were maintained in growth medium at 37 °C for 3 h. Subsequently, the absorbance at 490 nm was read on a microplate reader (Infinite M200 PRO; Tecan, Switzerland).

### Western blot analysis and immunoprecipitation (IP)

Mouse brain tissues or PC12 cells were collected and dissolved in lysis buffer. After centrifugation, the clarified lysates were collected, and the protein concentrations were measured using a bicinchoninic acid kit. Protein samples were used for electrophoresis and electrotransferred to nitrocellulose membranes (Millipore, Germany). Membranes were blocked with 5% milk in phosphate-buffered saline/0.1% Tween 20 (PBST) for 1 h to remove nonspecific binding. After incubation with the primary antibodies overnight at 4 °C, the membranes were washed and incubated with horseradish peroxidase-conjugated secondary antibody for 1 h at room temperature. Membrane blots were detected with the ECL chemiluminescence system as previously described [10] and detected on medical X-ray films. The film was scanned, and the density of the bands was analyzed using AlphaEase image analysis software (version 3.1.2). Primary antibodies against Ahi1 (sc-515382), GR (sc-56851), TFAM (sc-166965), COXIV (sc-376731) and Nup88 (sc-365868) (Santa Cruz Biotechnology, Delaware, CA, USA); COX-1 (PA5-26,688, Thermo Scientific, Rockford, IL, USA); β-actin (A5092) (Bimake, Houston, TX, USA); GAPDH (ab9485) and ATP5A (ab14748) (Abcam, Cambridge, MA, USA); were used. For immunoprecipitation, mitochondrial proteins (500 μg in 500 μl) were preincubated with Protein A/G agarose beads (20 μl) for 2 h. After gentle centrifugation, the supernatants were incubated with 5 μg mouse anti-Ahi1 antibody or mouse IgG at 4 °C overnight. The next day, Protein A/G agarose beads (50 μl) were added and incubated at room temperature for 2 h. The beads were centrifuged for 5 min at 2000×*g* at 4 °C and collected. After washing four times in lysis buffer, the beads were eluted by sample buffer containing sodium dodecyl sulfate. The samples were heated at 96 °C for 10 min for further Western blot analysis.

### Measurement of mtDNA levels

Genomic DNA was extracted using DNeasy Blood & Tissue Kits (Qiagen Inc., Hilden, Germany). Quantitative polymerase chain reaction (qPCR) and single-copy nuclear β-globin (Hbb) gene normalization were used to measure mitochondrial DNA copy number. Primers for mouse mtDNA (forward 5′-GCCCATGACCAACATAACTG-3′ and reverse 5′-CCTTGACGGCTATGTTGATG-3′) and mouse nuclear β-globin gene (Hbb) (forward 5′-AGGCAGAGGCAGGCAGAT-3′ and reverse 5′-GGCGGGAGGTTTGAGACA-3′) [50], rat mtDNA (forward 5′-ACACCAAGGTTAATGTAGC-3′ and reverse 5′-TTGAATCCATCTAAGCATT-3′), and rat nuclear β-globin gene (Hbb) (forward 5′-CAGTACTTTAAGTTGGAAACG-3′ and reverse 5′-ATCAACATAATTGCAGAGC-3′) were synthesized by GENEWIZ (Suzhou, China) [51].

### Quantitative polymerase chain reaction (qPCR)

Total RNA was extracted from mouse brains using the RNeasy Plus Mini kit according to the instructions. Reverse transcription was performed using the Transcript First Strand cDNA Synthesis Kit. Real-time PCR was performed in a 20 μl volume containing 50 ng cDNA,10 μl of 2XSYBR Green PCR Master Mix, and 1 μl primers (10 μM) by a 7500 real-time PCR machine (Applied Biosystems, USA). The primer sequences used in this study are listed in Additional file 1: Table S1.

### Chromatin immunoprecipitation (ChIP) assay

ChIP assays were performed according to the manufacturer's instructions using a ChIP assay kit (P2078, Beyotime Biotechnology). Briefly, fresh brain tissue samples were ground in liquid nitrogen and resuspended in phosphate-buffered saline (PBS) containing PMSF (ST506, Beyotime Biotechnology). After crosslinking in 1% formaldehyde, the reaction was stopped with glycine. The pellet was washed with PBS, lysed with SDS lysis buffer containing protease inhibitors and sonicated on ice to produce 200–500 bp DNA fragments. Then, 1% starting chromatin was saved as the input sample. After preclearance of the chromatin with Protein A + G Agarose beads, immunoprecipitation was performed with GR antibody (sc-56851), TFAM antibody (sc-166965) or normal IgG (12–370; Millipore) (as a negative control) overnight at 4 °C. The next day, 60 μl A + G agarose beads were added and incubated at 4 °C for 1 h. After washing, elution buffer was added to the precipitate, and DNA fragments were released from the immunoprecipitation complex by reverse crosslinking at 65 °C for 4 h. Thereafter, the DNA fragments were treated with protease K at 45 °C for 1 h. After phenol chloroform extraction, DNA fragments were precipitated with ethanol. Finally, the sample was dissolved in ddH_2_O for qPCR detection.

### Immunostaining

MitoTracker Red (50 nM) (#9082, Cell Signaling Technology Inc., Danvers, MA, USA) was added to the growing PC12 cell medium at 37 °C for 20 min. After incubation, the cells were fixed in ice-cold formalin and 1% Triton X-100 for 15 min, followed by rinsing in PBS three times for 5 min. Then, the cells were incubated with anti-Ahi1 antibody (sc-515382) at 4 °C overnight. The next day, the cells were incubated with the corresponding secondary antibody and DAPI at room temperature for 1 h. Images were digitized by a camera with a fluorescence microscope (Axio Scope A1, Carl Zeiss, Oberkochen, Germany).

### Statistical analysis

All data are expressed as the mean ± SEM. GraphPad Prism 8.0 (GraphPad Software, Inc.) was used for data analyses. Differences between two groups were determined by Student’s t test. The differences among groups were compared with one-way analysis of variance followed by Tukey’s multiple comparison tests or two-way analysis of variance followed by Bonferroni’s multiple comparisons post hoc test. P values < 0.05 were considered statistically significant.

## Results

### Ahi1 deficiency reduced cellular ATP and mitochondrial GR levels

**Fig. 1:**
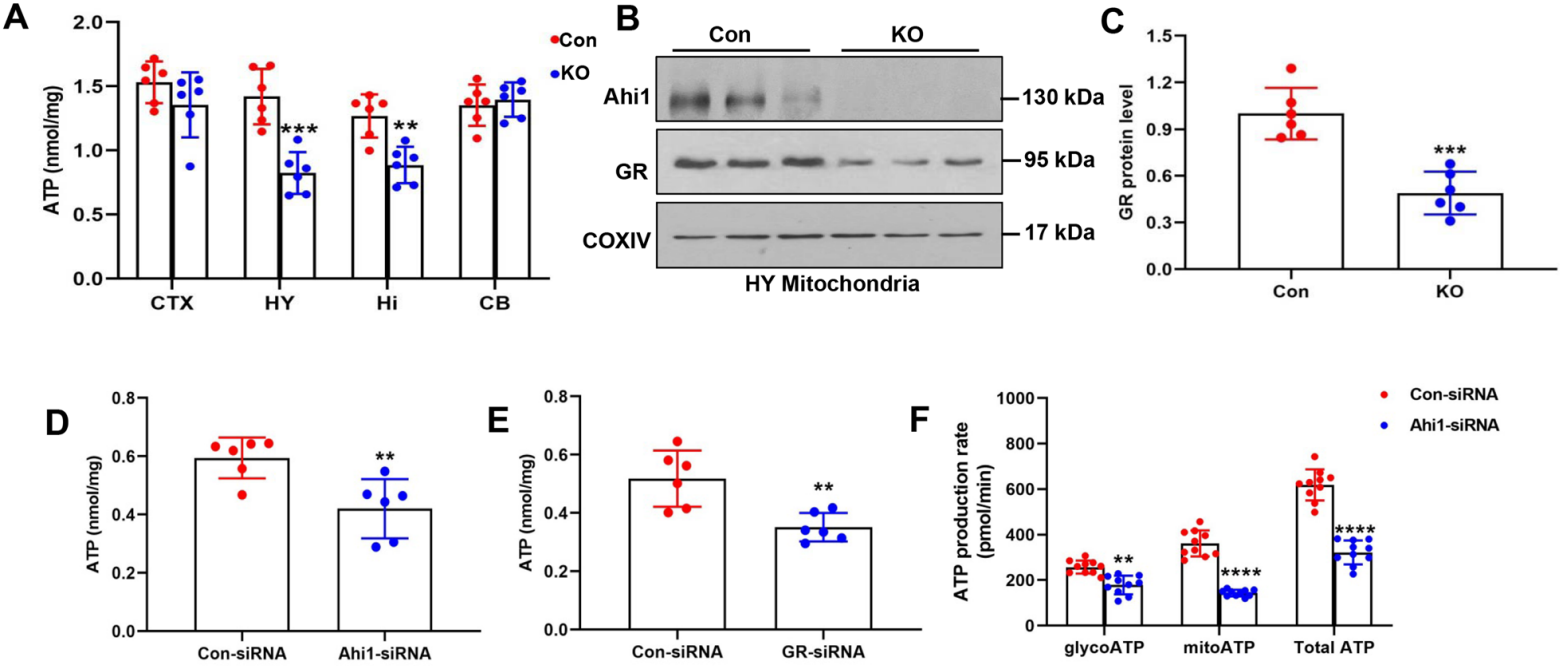
Ahi1 deficiency reduced cellular ATP and mitochondrial GR levels. ATP levels were measured in the cortex (CTX), hypothalamus (HY), hippocampus (Hi), and cerebellum (CB) in control mice and Ahi1 KO mice. N = 6 mice (**a**). Western blot analysis showed the expression of Ahi1 and GR in the mitochondria of hypothalamic tissues in control mice and Ahi1 KO mice. N = 6 mice (**b**, **c**). PC12 cells were transfected with Ahi1-siRNA or GR-siRNA, and ATP levels were detected 48 h after transfection. N = 6 cell samples (**d**, **e**). PC12 cells were transfected with Ahi1-siRNA for 48 h, and the levels of mitoATP, glycoATP, and total ATP production rates were detected. N = 10 cell samples (**f**). **P < 0.01, ***P < 0.001, ****P < 0.0001

Our previous study showed that Ahi1 knockout led to a depressive phenotype in mice [8, 9, 52], and depressive behaviors are associated with mitochondrial dysfunction [4–6]; therefore, we examined ATP levels in the brain. Before ATP measurement, depression-like behaviors in Ahi1 KO mice were also verified (Additional file 2: Fig. S1A–C). As expected, immobility time in the FST and the TST was significantly increased in Ahi1 KO mice, and sucrose consumption significantly decreased in Ahi1 KO mice. Furthermore, the ATP levels were determined in depression-related hypothalamic and hippocampal brain regions. We found that ATP levels in brain regions were significantly decreased in Ahi1 KO mice (Fig. 1a). Our previous studies have shown that Ahi1 binds to GR to regulate its nuclear translocation [10]. Total GR expression was reduced in the hypothalamus of Ahi1 KO mice (Additional file 3: Fig. S2A–B). Since GR is associated with mitochondrial function, ATP levels, and depressive behaviors [13, 14, 53], we examined GR levels in the hypothalamic mitochondria of Ahi1 KO mice. We first confirmed the purity of the mitochondrial fraction and found a significant fivefold enrichment of mitochondrial protein cytochrome c oxidase subunit I (COX-1) in the mitochondrial fraction (Additional file 4: Fig. S3A–B). Then, we investigated whether the mitochondrial fraction was contaminated by the nuclear or cell membrane fractions. We found that the levels of the nuclear pore protein marker Nup88 and the cytosolic marker glyceraldehyde 3-phosphate dehydrogenase (GADPH) or β-actin were very low in mitochondrial fractions (Additional file 4: Fig. S3A–B), indicating that the mitochondrial fraction had high purity. In addition, we found that GR levels were significantly reduced in the hypothalamus mitochondria of AHI1 KO mice (Fig. 1b, c). To examine the regulatory effect of Ahi1/GR on ATP levels, we knocked down Ahi1 or GR in PC12 cells and examined ATP levels. After Ahi1-siRNA or GR-siRNA transfection for 48 h, cellular ATP levels significantly decreased (Fig. 1d, e). As shown in Additional file 5: Fig. S4, Ahi1-siRNA did not decrease PC12 cell viability. In addition, Ahi1-siRNA transfection significantly decreased the production rates of mitoATP, glycoATP, and total ATP compared with Con-siRNA transfection (Fig. 1f), indicating that Ahi1 and GR levels in mitochondria were related to ATP levels and depression-like behaviors in mice.

### Mitochondrial Ahi1 interacted with GR and regulated ATP levels

**Fig. 2:**
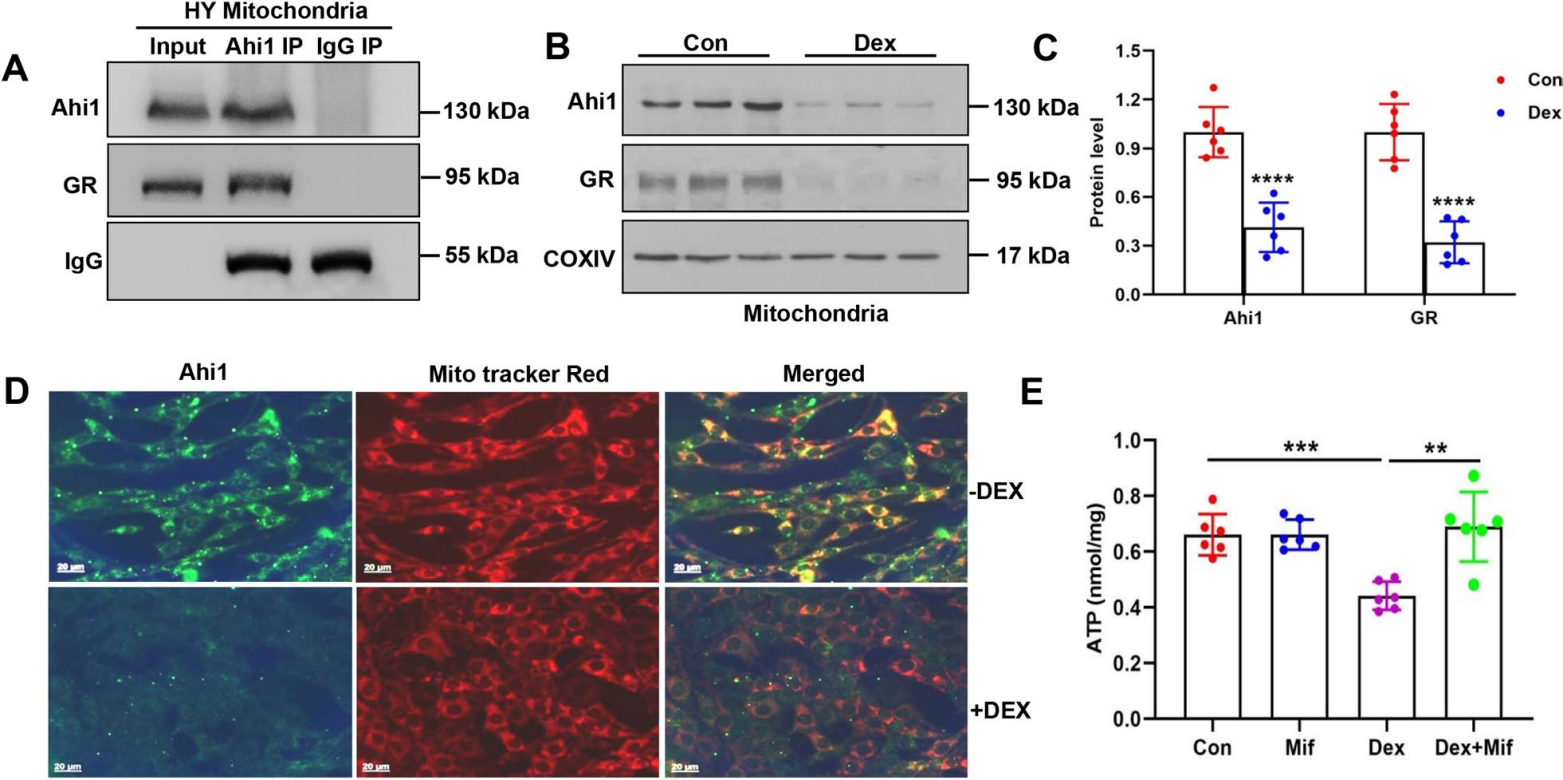
Mitochondrial Ahi1 interacted with GR and regulated ATP levels. Ahi1 antibody and control IgG antibody were used for immunoprecipitation in the mitochondria of mouse hypothalamus (HY), and Ahi1-interacting proteins were examined by Western blotting with anti-Ahi1 and anti-GR. Representative images are shown (**a**). PC12 cells were treated with Dex (20 μM) for 72 h. Ahi1 and GR were detected in the mitochondria by Western blotting. N = 6 cell samples (**b**, **c**). The expression of mitochondrial Ahi1 protein in PC12 cells treated with Dex for 72 h was detected by immunofluorescent staining (**d**). Scale bar = 20 μm. PC12 cells were treated with Dex (20 μM) with or without mifepristone (20 μM) for 72 h, and ATP content was assayed. N = 6 cell samples (**e**). **P < 0.01, ***P < 0.001, ****P < 0.0001

Our previous studies showed that Ahi1 interacts with GR and stabilizes each other in the cytoplasm [10]. In this study, we found that Ahi1 interacted with GR in mitochondria (Fig. 2a). Previous studies showed that dexamethasone (Dex, an agonist of GR) not only promotes the nuclear translocation of GR but also reduces GR in mitochondria [54]. Accordingly, we treated PC12 cells with Dex and examined the Ahi1 and GR levels in the mitochondria. Our results showed that Ahi1 and GR levels in mitochondria significantly decreased in PC12 cells after treatment with Dex for 72 h (Fig. 2b, c). Immunofluorescence staining also confirmed the reduction in Ahi1 protein expression in the mitochondria of Dex-treated PC12 cells (Fig. 2d). Consistently, ATP levels were significantly decreased in Dex-treated PC12 cells (Fig. 2e). Mifepristone is a GR antagonist that blocks the nuclear translocation of GR and facilitates the mitochondrial translocation of GR from the cytoplasm [54]. In line with this report, we also found that mifepristone inhibited the decrease in ATP levels caused by Dex treatment (Fig. 2e). Therefore, mitochondrial Ahi1 and GR may regulate ATP levels.

### The reduction in mitochondrial Ahi1/GR caused an increase in mtDNA copy numbers

**Fig. 3:**
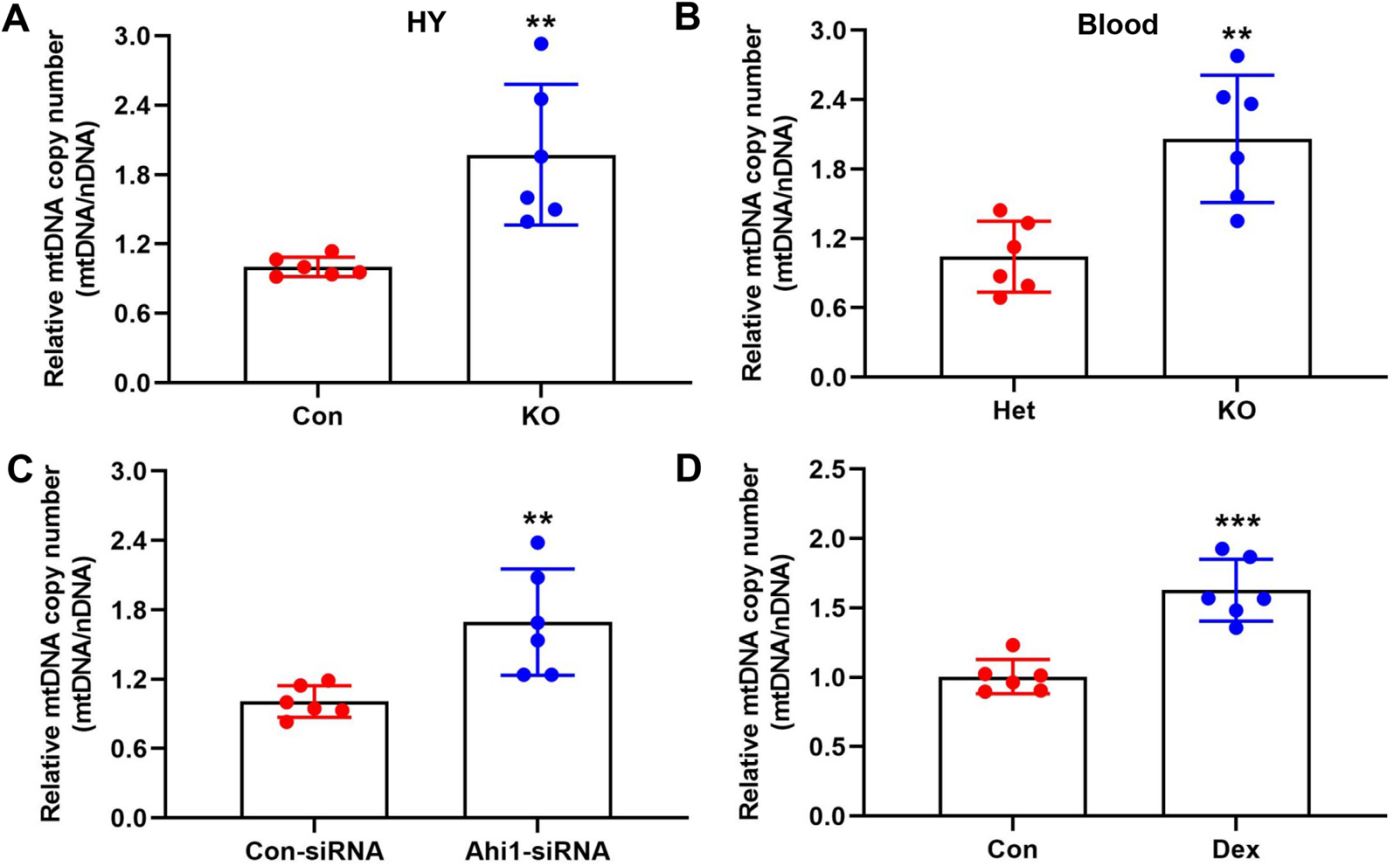
The reduction in mitochondrial Ahi1/GR caused an increase in mtDNA copy numbers. The mtDNA copy numbers in the hypothalamus (HY) and the blood from control mice and Ahi1 KO mice were detected by qPCR. N = 6 mice (**a**, **b**). PC12 cells were transfected with Ahi1-siRNA for 48 h, and the mtDNA copy number was detected by qPCR. N = 6 cell samples (**c**). PC12 cells were treated with Dex (20 μM) for 72 h, and the mtDNA copy number was detected by qPCR. N = 6 cell samples (**d**). **P < 0.01, ***P < 0.001

A growing number of studies have shown that oxidative phosphorylation is impaired in patients with major depressive disorder (MDD) or depressive mice, accompanied by an increase in mtDNA [4, 55, 56]. In our study, we examined mtDNA copy number in hypothalamic tissue and the blood of Ahi1 KO mice. We found that mtDNA copy numbers in the hypothalamus tissue and blood of Ahi1 KO mice were much higher than these in control mice (Fig. 3a, b). Ahi1 siRNA in PC12 cells significant increased mtDNA copy numbers (Fig. 3c). Similarly, Dex treatment also resulted in a significantly increase in mtDNA copy numbers (Fig. 3d). We also examined the expression of mitochondria-encoded genes and found that Ahi1 KO mice exhibited reduced levels of the mitochondria-encoded genes ND6 and ND4L, while Cox1 exhibited elevated levels (Additional file 6: Fig. S5). In addition, we found that the activities of citrate synthase and isocitrate dehydrogenase, two key enzymes of the tricarboxylic acid cycle, were unchanged in Ahi1 KO mice (Additional file 7: Fig. S6A–B). ATP synthase subunit α (ATP5A), which is related to ATP levels, was also unchanged in the hypothalamic tissue of Ahi1 KO mice (Additional file 7: Fig. S6C–D), implying that mitochondrial Ahi1/GR likely regulates the mtDNA-ATP pathway.

### Mitochondrial GR bound to the D-loop region of mtDNA and regulated mtDNA copy numbers with TFAM

**Fig. 4:**
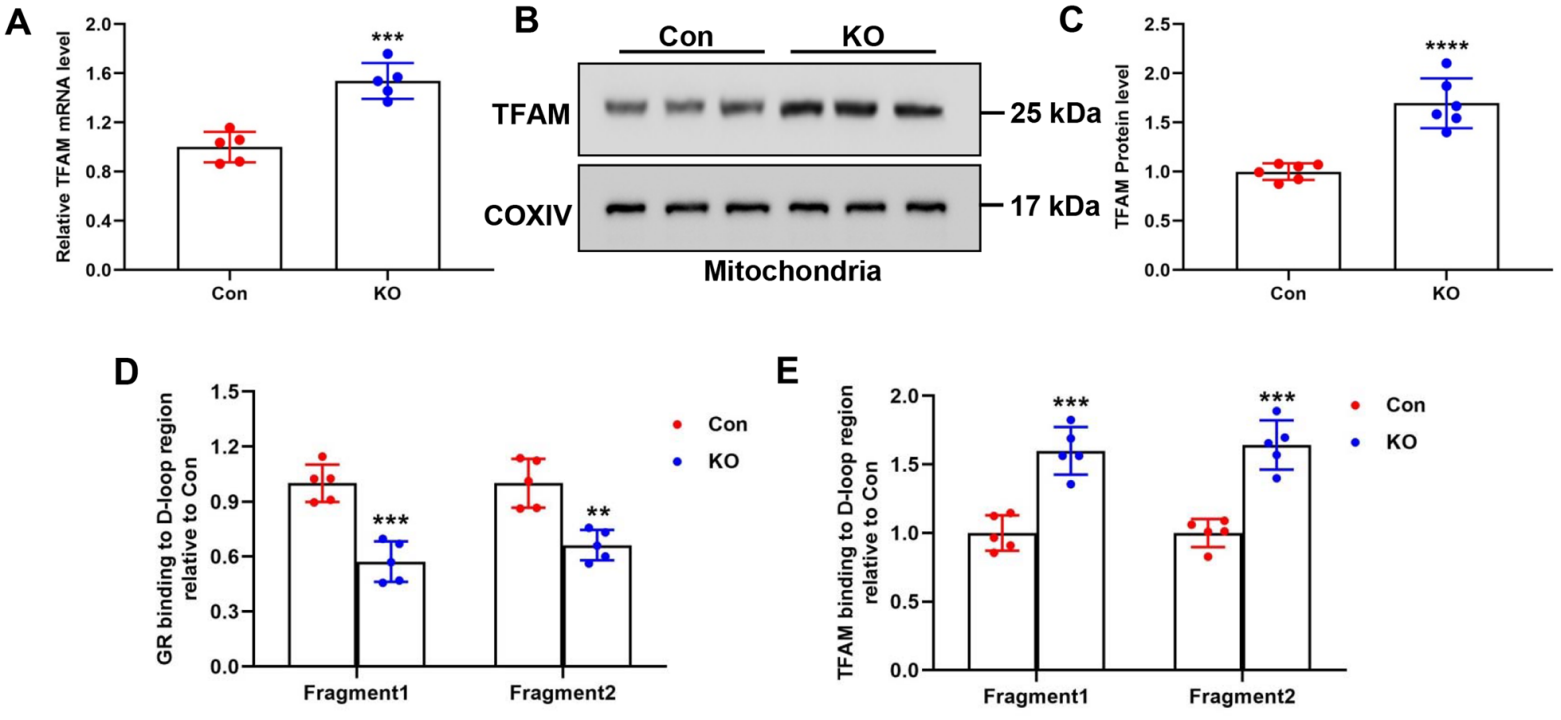
Mitochondrial GR and TFAM bound to the D-loop region of mtDNA and regulated mtDNA copy numbers. TFAM mRNA levels in the hypothalamus of Ahi1 KO mice were detected by qPCR. N = 5 mice (**a**). The TFAM in the hypothalamic mitochondria of the Ahi1 KO mice was detected by Western blotting. N = 6 mice (**b**, **c**). Relative GR and TFAM binding to the mtDNA D-loop region in the hypothalamus of Ahi1 KO mice was detected by ChIP/qPCR. N = 5 mice (**d**, **e**). **P < 0.01, ***P < 0.001, ****P < 0.0001

Many molecular and genetic models have demonstrated a direct correlation between TFAM and mtDNA levels [19–21]. Previous studies also showed that TFAM can bind to the mitochondrial D-loop region, which is involved in regulating mtDNA replication [18, 57]. Strikingly, GR can also bind the D-loop region [11, 12, 58]. Therefore, we speculated that mitochondrial GR and TFAM may coordinately regulate mtDNA copy numbers. As shown in Fig. 4a, TFAM mRNA levels significantly increased in the hypothalamus of Ahi1 KO mice. Interestingly, TFAM protein levels also significantly increased in the mitochondrial fraction of the hypothalamus (Fig. 4b, c). To further examine the binding of GR or TFAM to the D-loop region of mtDNA, we found that GR binding to the mtDNA D-loop region significantly reduced in the hypothalamic region of Ahi1 KO mice (Fig. 4d). In contrast, TFAM binding to the mtDNA D-loop region significantly increased (Fig. 4e).

### Exogenous ATP had rapid antidepressant-like effects in Ahi1 KO mice

**Fig. 5:**
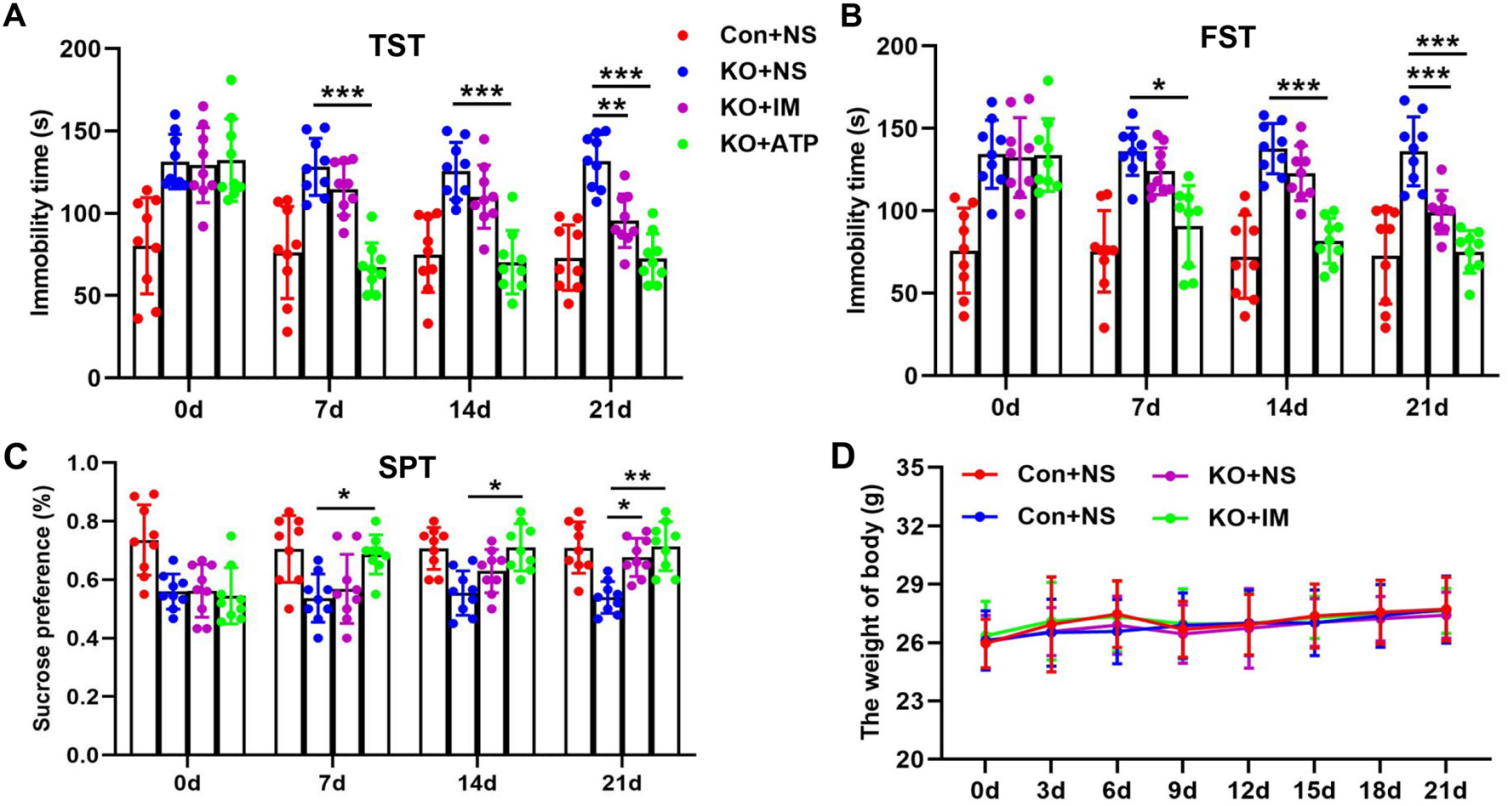
Exogenous ATP had rapid antidepressant-like effects in Ahi1 KO mice. Ahi1 KO mice were intraperitoneally injected with ATP (125 mg/kg) and the antidepressant imipramine (IM, 20 mg/kg) for 3 weeks. Immobility time in the tail suspension test (TST) and forced swimming test (FST) were examined in control mice and Ahi1 KO mice. N = 9 mice (**a**, **b**). A sucrose preference test was performed, and the percentage of sucrose consumed in the total drinking water was calculated. N = 9 mice (**c**). Body weight was recorded from the day of administration of Ahi1 KO mice for 3 weeks, and a growth curve of body weight was drawn. N = 9 mice (**d**). *P < 0.05, **P < 0.01, ***P < 0.001

Previous studies have shown that intraperitoneal injection of ATP in CSDS mice can have a significant antidepressant-like effect [6]. To test whether ATP could induce an antidepressant-like effect in Ahi1 KO mice, Ahi1 KO mice were treated with an intraperitoneal injection of ATP and the antidepressant imipramine as a control for 3 weeks. The results showed that treatment with the classical antidepressant imipramine for 3 weeks significantly improved depression-like behaviors in Ahi1 KO mice (Fig. 5a–c). In contrast, ATP treatment for one week significantly improved depression-like behaviors in Ahi1 KO mice (Fig. 5a–c) but was ineffective in imipramine treatment, suggesting that ATP is a faster-acting antidepressant. In addition, we also detected the effects of ATP and imipramine on the bodyweight of Ahi1 KO mice. By weighing, we found that treatment with either treatment of ATP or imipramine for 3 weeks had no effect on the bodyweight of Ahi1 KO mice (Fig. 5d). These results thereby support the hypothesis that exogenous ATP induces rapid antidepressant-like effects in Ahi1 KO mice.

### Regular exercise increased mitochondrial Ahi1/GR levels and ATP levels in the brains of Dex-treated mice

**Fig. 6:**
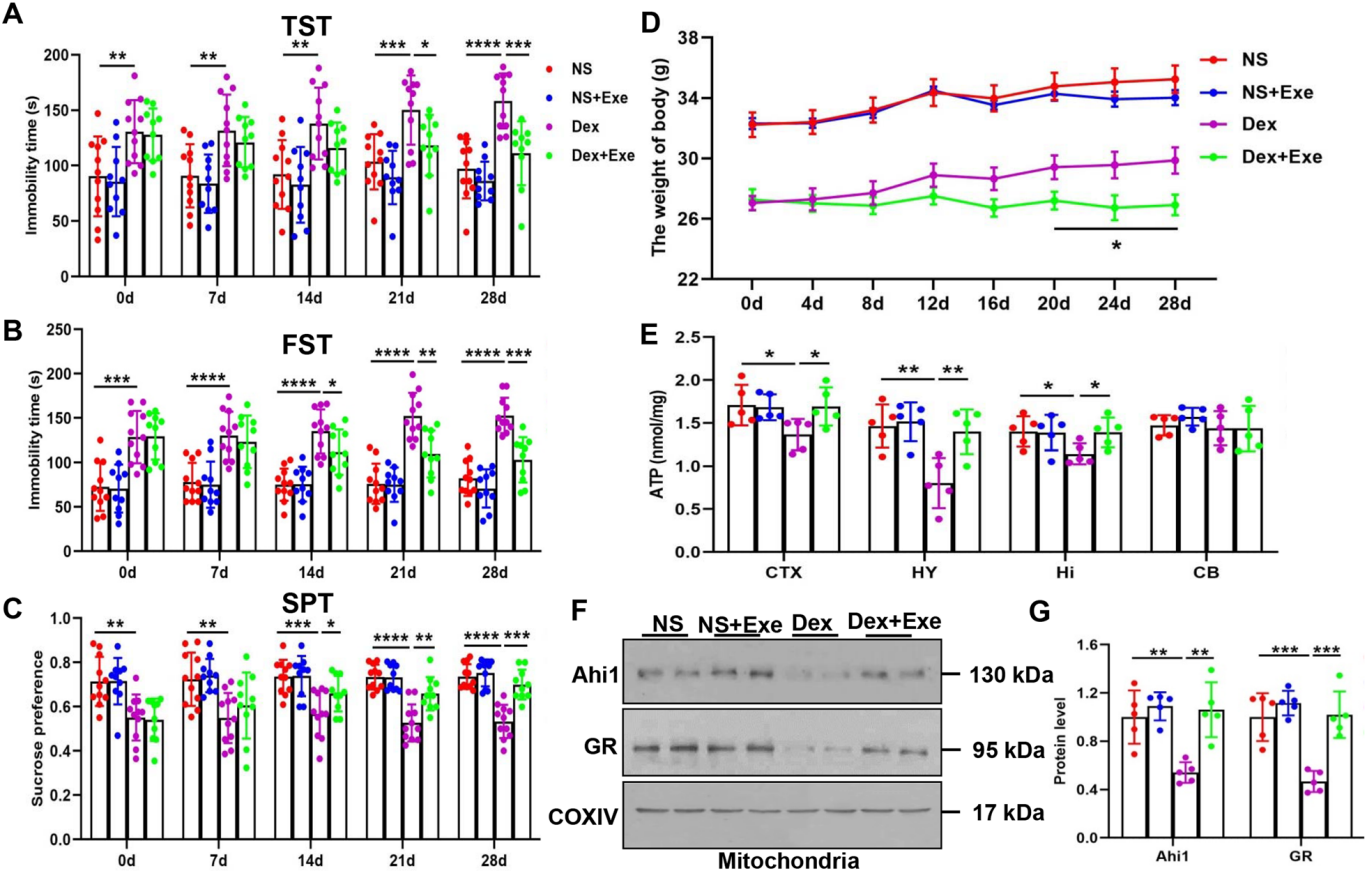
Regular exercise increased mitochondrial Ahi1 and GR levels and ATP levels in the brains of Dex-treated mice. Mice were divided into NS, NS + Exe, Dex, and Dex + Exe groups. Regular exercise lasted for 4 weeks, and behaviors were measured every week. Immobility time in the tail suspension test (TST) and forced swimming test (FST) were examined. N = 10–11 mice (**a**, **b**). A sucrose preference test was performed and the percentage of sucrose consumed in the total drinking water was calculated. N = 10–11 mice (**c**). The weights of the mice were recorded for 4 weeks, and a weight growth curve was drawn. N = 10–11 mice (**d**). After 4 weeks of exercise, ATP levels were measured in the cortex (CTX), hypothalamus (HY), hippocampus (Hi), and cerebellum (CB) of four groups of mice. N = 5 mice (**e**). Western blot analysis showed the expression of Ahi1 and GR in the mitochondria of hypothalamic tissue in the four groups. N = 5 mice (**f**, **g**). *P < 0.05, **P < 0.01, ***P < 0.001, ****P < 0.0001

A growing body of clinical data and animal studies suggest that physical exercise can improve depressive behaviors [34, 38]. To examine whether regular exercise attenuates depressive behaviors by alleviating mitochondrial functions, we induced a depressive mouse model by subcutaneous Dex injection for 21 days and identified depression-like behaviors by behavioral tests (Additional file 8: Fig. S7A–C). A significant decrease in ATP levels was found in the hypothalamus and hippocampus in Dex-treated mice (Additional file 8: Fig. S7D). In addition, the body weight was significantly lower in the Dex-induced depressive mice group than in the NS group (Additional file 8: Fig. S7E). The test mice were divided into four groups: NS, NS + Exe, Dex and Dex + Exe. The exercise group was subjected to 4 weeks of exercise with weekly behavioral tests. We found that mice in the Dex + Exe group showed lower immobility time in the FST and the TST than mice in the Dex group (Fig. 6a, b) and that mice in the Dex + Exe group preferred a higher sucrose content in the SPT than mice in the Dex group (Fig. 6c). In addition, the bodyweight of mice in the Dex + Exe group was lower than that in the Dex group (Fig. 6d). Since ATP levels are linked with depression, we tested whether exercise improves ATP levels in mice. As expected, ATP levels were significantly decreased in depression-related regions such as the hypothalamus, hippocampus, and cortex in the Dex group compared with the NS group (Fig. 6e). In contrast, the Dex + Exe group showed higher ATP levels than the Dex group (Fig. 6e). In addition, the levels of mitochondrial Ahi1 and GR were significantly decreased in Dex-induced depressive mice but significantly increased in the Dex + Exe group compared with the Dex group (Fig. 6f, g), indicating that regular exercise increased mitochondrial Ahi1 and GR levels and ATP levels in the brains of Dex-treated mice.

### Regular exercise increased ATP levels in control mice but not in Ahi1 KO mice under stress

**Fig. 7:**
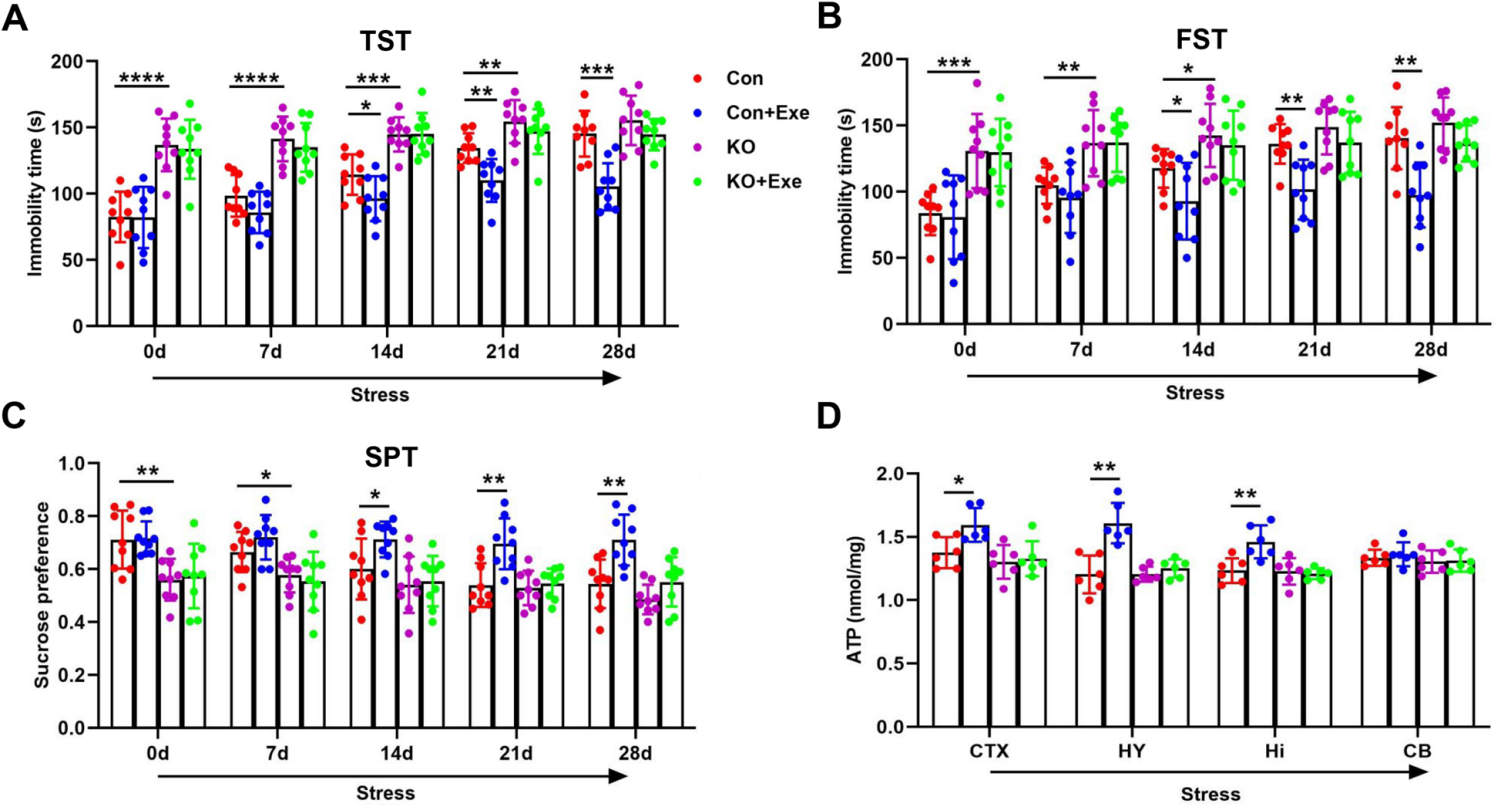
Regular exercise increased ATP levels in control mice but not in Ahi1 KO mice under stress. Con mice and Ahi1 KO mice were divided into four groups: Con, Con + Exe, KO, and KO + Exe. Mice were placed in 50 ml tubes for space restriction from 9:00 to 11:00 each day. Immobility time in the tail suspension test (TST) and forced swimming test (FST) were examined. N = 9 mice (**a**, **b**). A sucrose preference test was performed and the percentage of sucrose consumed in the total drinking water was calculated. N = 9 mice (**c**). All mice received spatial restraint stress. After 4 weeks of exercise, ATP levels were measured in the cortex (CTX), hypothalamus (HY), hippocampus (Hi), and cerebellum (CB) in the four groups. N = 6 mice (**d**). *P < 0.05, **P < 0.01, ***P < 0.001, ****P < 0.0001

To further substantiate that exercise regulates ATP levels and depression-like behavior through the mitochondrial Ahi1/GR complex. Control mice and Ahi1 KO mice for the test were divided into four groups: Con, Con + Exe, KO, and KO + Exe groups. All mice were subjected to the spatial restraint stress each day as described previously [10]. Under stress, the control group gradually showed depression-like behaviors, while no depression-like behaviors were observed in the Con + Exe group compared with the control group. In contrast, no significant difference was found in the KO + Exe group compared with the KO group (Fig. 7a–c). Moreover, we also examined the effect of 4 weeks of exercise on ATP levels in Ahi1 KO mice under spatial restraint stress. Under stress, compared with the control group, the Con + Exe group exhibited increased levels of ATP in depression-related brain regions of the hypothalamus, hippocampus, and cortex, but not in the cerebellum. In contrast, the alterations in ATP levels were undetectable in the KO + Exe group compared with the KO group (Fig. 7d), thereby suggesting that regular exercise improves depression-like behaviors and elevates ATP levels in the control mice but does not impact Ahi1 KO mice under stress.

## Discussion

Mitochondria are organelles that widely exist in almost every eukaryotic cell, participate in a variety of cell functions and are very important for cell survival and death [26]. The function of mitochondria mainly includes the production of cellular ATP, involvement in the synthesis of key metabolites, regulation of apoptosis, calcium buffering, and the primary source of endogenous reactive oxygen species [59–61]. Accumulating evidence suggests that mitochondrial dysfunction is closely related to neuropsychiatric diseases, including bipolar disorder (BD), schizophrenia (SZ), and depression [27, 42, 62, 63], but the regulatory mechanisms of mitochondrial dysfunction remain unclear. In this study, we found that Ahi1 regulated the mitochondrial location of GR and further controlled mitochondrial DNA copy numbers as well as ATP levels by modulating GR binding to the D-loop control region of mitochondrial DNA. However, it is unclear whether ATP reduction in the brains of Ahi1 KO mice is mainly attributed to reduced ATP production or increased energy demand.

GR is an important gene involved in the stress response [64]. Increasing evidence suggests that GR can bind to the D-loop control region of mitochondrial DNA to regulate gene expression [11, 12]. Interestingly, TFAM can also bind to the D-loop control region of mitochondria and positively regulate mitochondrial replication and transcription [16, 17, 21]. In the present study, we found that Ahi1 KO caused a decrease in ATP and GR levels in the mitochondria (Fig. 1a) and an increase in mtDNA copy numbers (Fig. 3a) in mouse hypothalamic tissues. In addition, mtDNA was also significantly increased in Ahi1-siRNA transfected or Dex-treated PC12 cells (Fig. 3c, d). Notably, the TFAM levels were significantly increased in the mitochondrial fraction of Ahi1 KO mice (Fig. 4b, c). Through ChIP experiments, we found that the binding of GR to the mtDNA D-loop region was significantly reduced in the hypothalamic region of Ahi1 KO mice; in contrast, the binding of TFAM to the mtDNA D-loop region was significantly increased (Fig. 4d, e). Thus, we speculated that mitochondrial GR and TFAM may compete with the D-loop region of mtDNA to regulate mtDNA copy numbers. Consistent with this finding, energy production was reduced in stressed mice with elevated mtDNA levels [4]. Therefore, this is the first study to demonstrate that mitochondrial Ahi1/GR coordinately regulates mtDNA copy numbers by the binding to the D-loop region of mtDNA with TFAM.

We found that Ahi1 KO mice showed reduced expression of the mitochondria-encoded genes ND-6 and ND-4L, while Cox1 expression was elevated (Additional file 6: Fig. S5). Although Ahi1 KO causes GR nuclear translocation, increased nuclear GR does not affect nuclear encoded gene products, such as CS and IDH activities and ATP5A levels [10]. This suggests that Ahi1 KO affects mitochondria-encoded genes but not nuclear-encoded mitochondrial genes. In this study, we also found that Ahi1 KO resulted in elevated mtDNA copy number and decreased mitochondrial gene expression (Additional file 6: Fig. S5). These inconsistencies in mtDNA copy number and mitochondrial gene expression have been reported previously [65, 66]. However, it is unclear why high mtDNA copy numbers are related to low mitochondrial gene expression. Our present study indicated that Ahi1 KO causes increased mtDNA and TFAM with decreased ATP levels. Nevertheless, we cannot rule out the possibility that Ahi1 KO impairs mitochondrial function independently of GR/D-loop effects because previous studies have verified that GR binding to the mtDNA promoter seems to be inversely correlated with mtDNA 5mC and 5hmC modifications [67]. The expression of mtDNA is affected by methylation and hydroxylation of mtDNA [68]. Therefore, the increase in TFAM and mtDNA is also likely to be a compensatory response to the mitochondrial damage caused by Ahi1 KO, independent of the competition of TFAM and GR for the binding of the D-loop of mtDNA.

A significant body of studies has shown that regular physical exercise improves depressive states [34, 38], but the mechanism of this effect remains unclear. Exercise can increase ATP levels in skeletal muscle, thereby exerting an antidepressant effect [69]. In addition, intraperitoneal injection of ATP in CSDS mice also resulted in a rapid antidepressant-like effect [6]. In line with this, we observed that ATP could rapidly improve depression-like behavior in Ahi1 KO mice compared to imipramine treatment (Fig. 5a–c). This suggests that ATP may be a faster-acting antidepressant. In our studies, we found that exercise significantly improved depression-like behaviors (Fig. 6a–c) and increased mitochondrial Ahi1/GR levels and ATP levels in the hypothalamic tissues of Dex-induced mice (Fig. 6e, f). However, compared with the KO group, exercise did not change ATP levels or improve depressive behaviors in Ahi1 KO mice under stress (Fig. 7a–d), indicating that exercise regulates ATP levels and improves depression-like behavior in mice through the mitochondrial Ahi1/GR/mtDNA pathway.

## Conclusions

In this study, we demonstrated that the mitochondrial Ahi1/GR and TFAM coordinately regulate mtDNA copy numbers and ATP levels by binding to the D-loop region of mtDNA. Regular exercise increases mitochondrial Ahi1/GR levels and ATP levels and improves depression-like behaviors in mice.

## Supplementary Information

**Additional file 1: Table S1.** Primers used in this study

**Additional file 2: Fig. S1.** Ahi1 KO mice showed depression-like behaviors.

**Additional file 3: Fig. S2.** Total GR expression was reduced in the hypothalamus of Ahi1 KO mice.

**Additional file 4: Fig. S3.** The purity of the mitochondrial fraction was examined in the hypothalamus.

**Additional file 5: Fig. S4.** Ahi1 knockdown did not alter cell viability.

**Additional file 6: Fig. S5.** Mitochondrial gene expression in the hypothalamus of Ahi1 KO mice was examined.

**Additional file 7: Fig. S6.** Citrate synthase activity, isocitrate dehydrogenase activity, and ATP5A content were unchanged in Ahi1 KO mice.

**Additional file 8: Fig. S7.** Dexamethasone induced depression-like behavior in mice.

**Additional file 9: Fig. S8.** ATP stability was evaluated by an ATP kit.

## Data Availability

All data generated or analyzed during this study are included in this published article.

## Abbreviations

AHI1: Abelson helper integration site 1
mtDNA: Mitochondrial DNA
KO: Knockout
GR: Glucocorticoid receptors
TFAM: Mitochondrial transcriptional factor A
CSDS: Chronic social defeat stress
FST: Forced swim test
TST: Tail suspension test
SPT: Sucrose preference test
IP: Immunoprecipitation
qPCR: Quantitative polymerase chain reaction
ChIP: Chromatin immunoprecipitation
COX-1: Cyclooxygenase-1
Dex: Dexamethasone
MDD: Major depressive disorder
