# Supplementary material for: The mitochondrial Ahi1/GR participates the regulation on mtDNA copy numbers and brain ATP levels and modulates depressive behaviors in mice
Source: Cell Commun Signal. 2023 Jan 23;21:21. doi: 10.1186/s12964-022-01034-8 (PMC9869592; doi:10.1186/s12964-022-01034-8)
Supplement: Supplementary file 2 — Additional file 1: Table S1. Primers used in this study [file 12964_2022_1034_MOESM2_ESM.docx]

**The mitochondrial Ahi1/GR participates the regulation on mtDNA copy numbers and brain ATP levels and modulates depressive behaviors in mice**

Bin Wang^1,2^, Haixia Shi^2^, Bo Yang^3^, Zhigang Miao^2^, Miao Sun^1^,

Hao Yang^1^, Xingshun Xu^2,4,5^

^1^Department of Fetology, the First Affiliated Hospital of Soochow University, Suzhou, 215006, China; ^2^Institute of Neuroscience, Soochow University, Suzhou, 215123, China; ^3^Department of Anesthesiology, The Second Affiliated Hospital of Soochow University, Suzhou, 215004, China; ^4^Department of Neurology, the First Affiliated Hospital of Soochow University, Suzhou, 215006, China; ^5^Jiangsu Key Laboratory of Neuropsychiatric Diseases, Soochow University, Suzhou, Jiangsu 215123, China.

These authors contributed equally: Bin Wang, Haixia Shi, Bo Yang.

***Correspondence authors:**

**Xingshun Xu MD, PhD**

Department of Neurology, the First Affiliated Hospital of Soochow University, Suzhou, China, 215006, Email: xingshunxu@suda.edu.cn, Telephone: 86-512-65883252

**Hao Yang, PhD**

Department of Fetology, the First Affiliated Hospital of Soochow University, Suzhou, China, 215006, Email: yanghao.71_99@yahoo.com, Telephone: 86-512-67786096

**Additional file**

**Additional files 1**

**Table S1 Primers used in this study.**

| **Gene name** | **Forward** | **Reverse** |
| --- | --- | --- |
| TFAM | CGGCTCAGGGAAAATTGAAGC | TTCAGCCATCTGCTCTTCCC |
| GAPDH | CATGCCTTCCGTGTTCCTA | CTTCACCACCTTCTTGATGTCATC |
| D-loop fragment 1  D-loop fragment 2  ND-1  ND-2  ND-3  ND-4  ND-4L  ND-5  ND-6  Cytb  Cox1  Cox2  Cox3  ATP6  ATP8 | AATCAATGGTTCAGGTCA  CTCGATGGTATCGGGTCT  GAGCCCGGTAATCGCATAA  CGTCACACAAGCAACAGCCTCAAT  TGCGGATTCGACCCTACAAG  AATCGCCTACTCCTCAGTTAGCCA  TGCCATCTACCTTCTTCAACCTCACC  ATAGCCTGGCAGACGAACAAGACA  GTTGGAGTTATGTTGGAAGGAGGG  ATTCCTTCATGTCGGACGAG  CCCAAAACCGACAAGGACTAC  ATCGAGCGGGGAAAGACATAC  TAACCCTTGGCCTACTCACC  CTCACTTGCCCACTTCCTTC  AACATTCCCACTGGCACCTTC | ACGGAGGATGGTAGATTA  GCCTTAGGTGATTGGGTT  GATAGGTGGCACGGAGAAT  TGTGCAGTGGGATCCCTTGAGTTA  TGCTCATGGTAGTGGAAGTAGA  AGGAGTGATGATGTGAGGCCATGT  TGCCTTCCAGGCATAGTAATGTGG  AATTAGTAGGGCTCAGGCGTTGGT  CCGCAAACAAAGATCACCCAGCTA  ACTGAGAAGCCCCCTCAAAT  ACATAAGTCGCAATGGCTTCTT  TGATGGTACAGCCACCTTAGG  AATAGGAGTGTGGTGGCCTTG  GTAAGCCGGACTGCTAATGC  TATTGTTGGGGTAATGAATGAGGC |

**Additional files 2**

**Fig. S1. Ahi1 KO mice showed depression-like behaviors.** Behavioral tests were performed in 2–3-month-old mice. Immobility time in the tail suspension test (TST) and forced swimming test (FST) were examined in control mice and Ahi1 KO mice. N=10 mice (**A**-**B**). A sucrose preference test was performed, and the percentage of sucrose consumed in the total drinking water was calculated. N=10 mice (**C**). ***P < 0.001, ****P < 0.0001.

**Additional files 3**

**Fig. S2. Total GR expression was reduced in the hypothalamus of Ahi1 KO mice.** Total GR expression in the hypothalamus of control and Ahi1 KO mice was examined by Western blotting (**A**). Total GR was analyzed and compared between the two groups (**B**). N=3 mice, **P <0.01.

**Additional files 4**

**Fig. S3. The purity of the mitochondrial fraction was examined in the hypothalamus.** The mitochondrial marker COX1 was enhanced 5-fold in the mitochondrial fraction. N=3 mice (**A**-**B**). The mitochondrial fraction showed a low content of the cytosol marker GAPDH or β-actin. N=3 mice (**A**-**B**). The absence of the nuclear pore protein marker Nup88 was found in the mitochondrial fraction. N=3 mice (**A**-**B**). **P <0.01, ****P < 0.0001.

**Additional files 5**

**Fig. S4. Ahi1 knockdown did not alter cell viability.** PC12 cells were transfected with Ahi1-siRNA or control siRNA and cell viability was determined at 48 h after transfection by the MTS assay. N=8 cell samples.

**Additional files 6**

**Fig. S5. Mitochondrial gene expression in the hypothalamus of Ahi1 KO mice was examined.** Fresh hypothalamus tissues were collected and total RNA was extracted for the detection of mitochondrial gene expression. The mRNA levels of 13 mitochondrial genes were determined by quantitative PCR. Of these genes, ND6 and ND-4L were significantly downregulated, and Cox1 was upregulated. N=5 mice. *P <0.05.

**Additional files 7**

**Fig. S6. Citrate synthase** **activity, isocitrate dehydrogenase activity, and ATP5A content were unchanged in Ahi1 KO mice.** The activities of citrate synthase and isocitrate dehydrogenase in the hypothalamus of Ahi1 KO mice and control mice were detected by ELISA. N=5 mice (**A**-**B**). ATP5A in the hypothalamus of Ahi1 KO mice and control mice was detected by Western blotting. N=6 mice (**C**-**D**).

**Additional files 8**

**Fig. S7. Dexamethasone induced depression-like behavior in mice.** Two -month-old male ICR mice were exposed to subcutaneous injections of Dex (1 mg/kg) or normal saline (NS) for 21 days. Immobility time in the tail suspension test (TST) and forced swimming test (FST) were examined in control mice and Ahi1 KO mice. N=21 mice (**A**-**B**). A sucrose preference test was performed, and the percentage of sucrose consumed in the total drinking water was calculated. N=21 mice (**C**). ATP levels were measured in the cortex (CTX), hypothalamus (HY), hippocampus (Hi), and cerebellum (CB) in the NS group and Dex-induced depressed group. N=6 mice (**D**). The body weights of Dex-induced depressive mice were recorded for 3 weeks, and a growth curve of body weight was drawn. N=21 mice (**E**). *P <0.05, **P <0.01, ****P < 0.0001.

**Additional files 9**

**Fig. S8. ATP stability was evaluated by an ATP kit.** PC12 cells were treated with EtOH (con) or 30 μM FCCP for 30 min. Total ATP levels in the cell extract were measured. N=8 cell samples (**A**). The hypothalamus tissues were stored at -80 ℃ for one month. ATP levels were measured in fresh and frozen tissues. N=7 mice (**B**). Fresh hypothalamus tissues were placed on ice for different times and ATP levels were measured by an ATP kit. N=6 mice (**C**). ****P < 0.0001.
