# Supplementary figures and images for: The mitochondrial Ahi1/GR participates the regulation on mtDNA copy numbers and brain ATP levels and modulates depressive behaviors in mice
Source: Cell Commun Signal. 2023 Jan 23;21:21. doi: 10.1186/s12964-022-01034-8 (PMC9869592; doi:10.1186/s12964-022-01034-8)

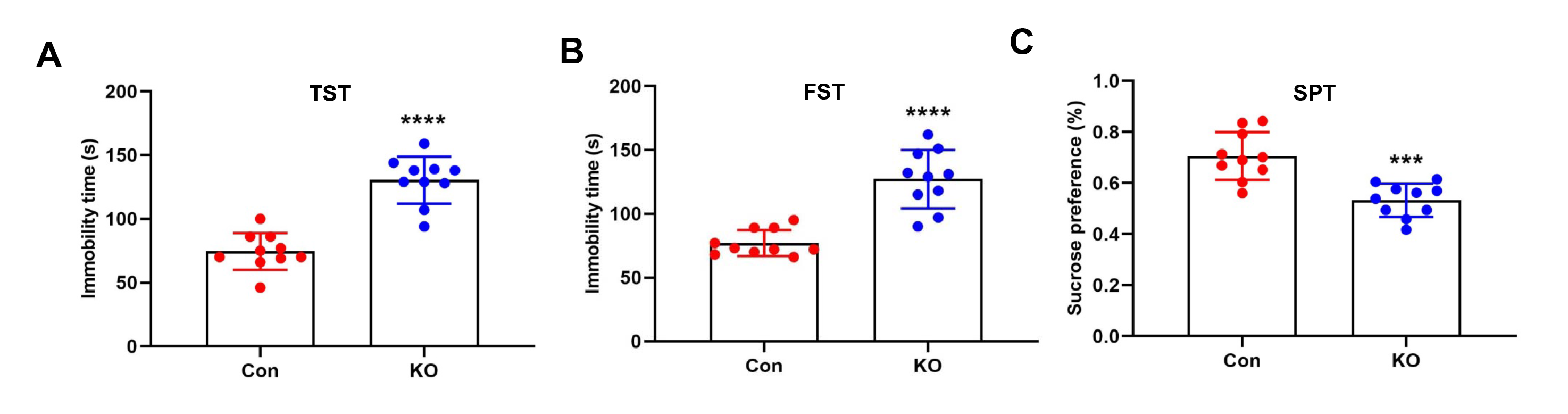

Supplement: Supplementary file 3 — Additional file 2: Fig. S1. Ahi1 KO mice showed depression-like behaviors. [file 12964_2022_1034_MOESM3_ESM.tif]

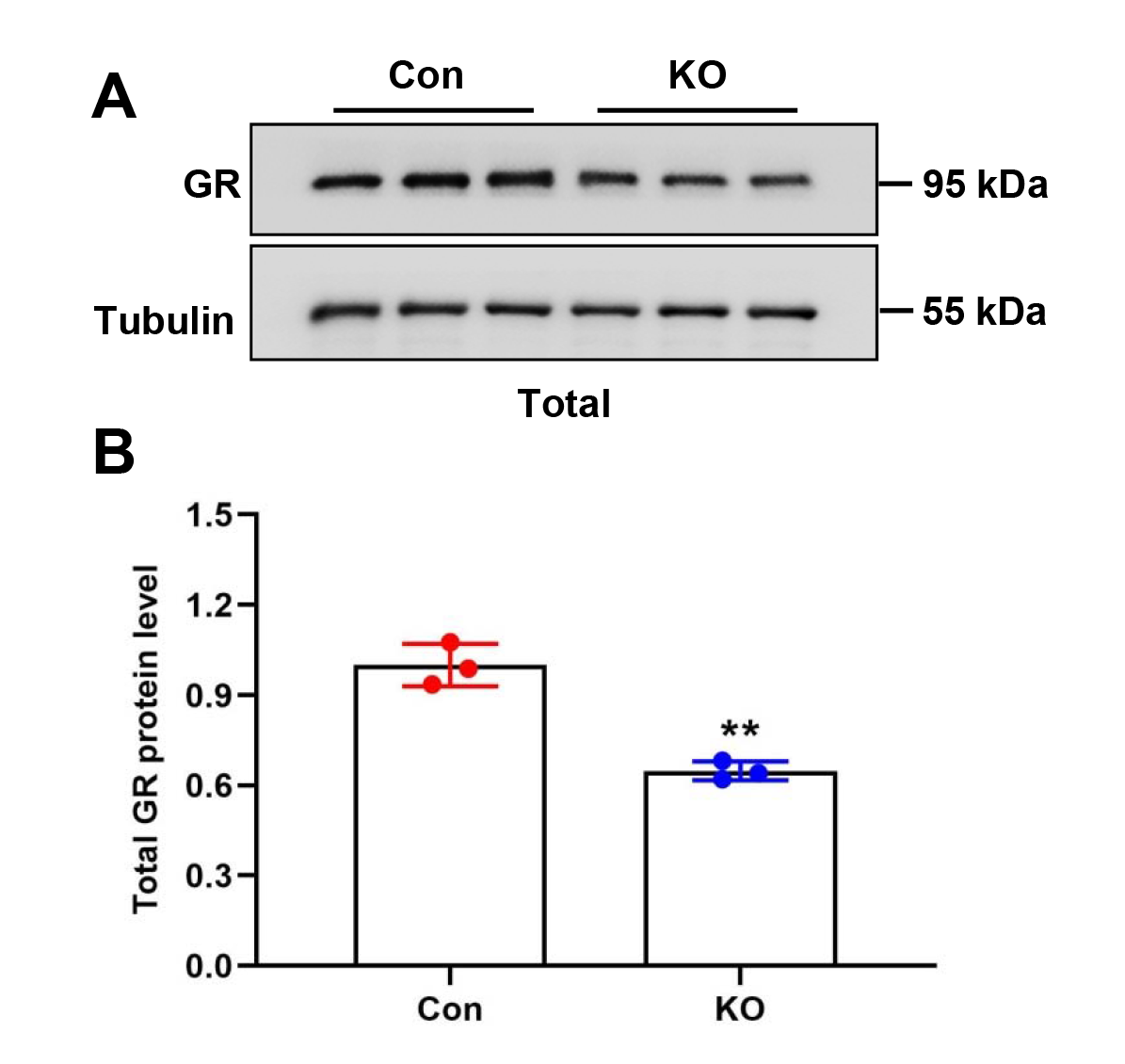

Supplement: Supplementary file 4 — Additional file 3: Fig. S2. Total GR expression was reduced in the hypothalamus of Ahi1 KO mice. [file 12964_2022_1034_MOESM4_ESM.tif]

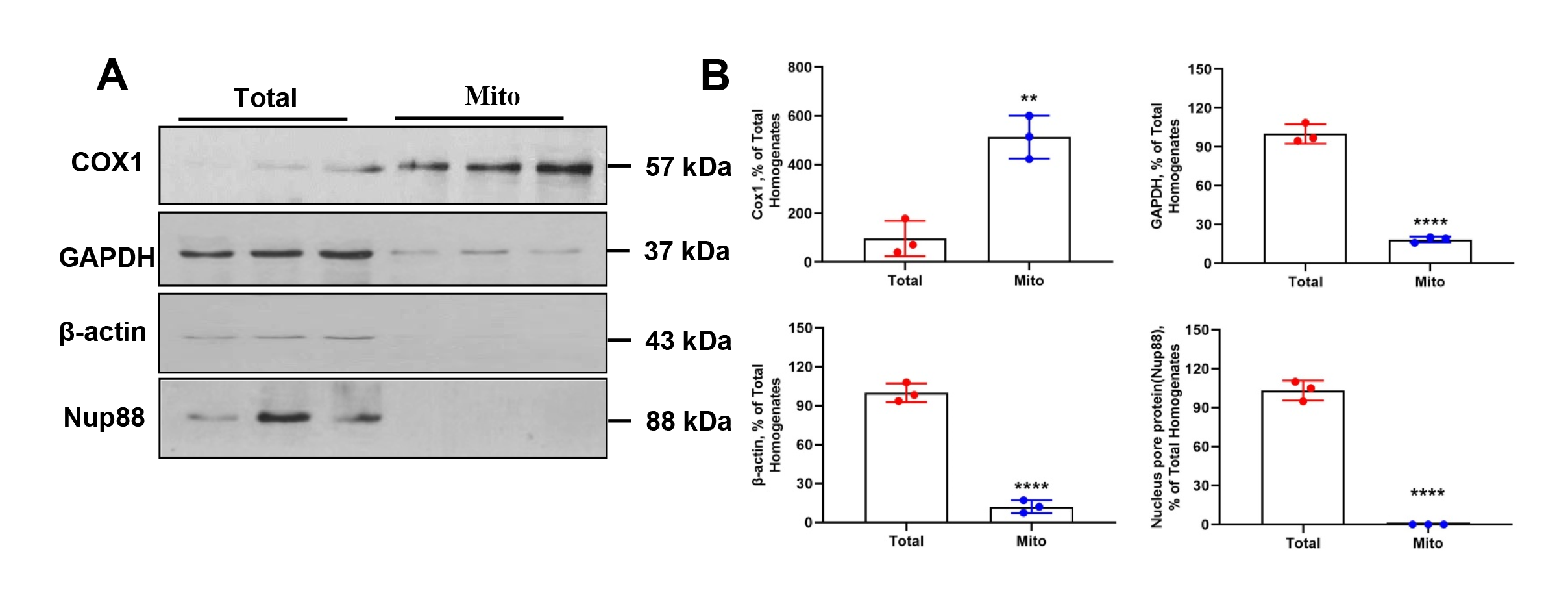

Supplement: Supplementary file 5 — Additional file 4: Fig. S3. The purity of the mitochondrial fraction was examined in the hypothalamus. [file 12964_2022_1034_MOESM5_ESM.tif]

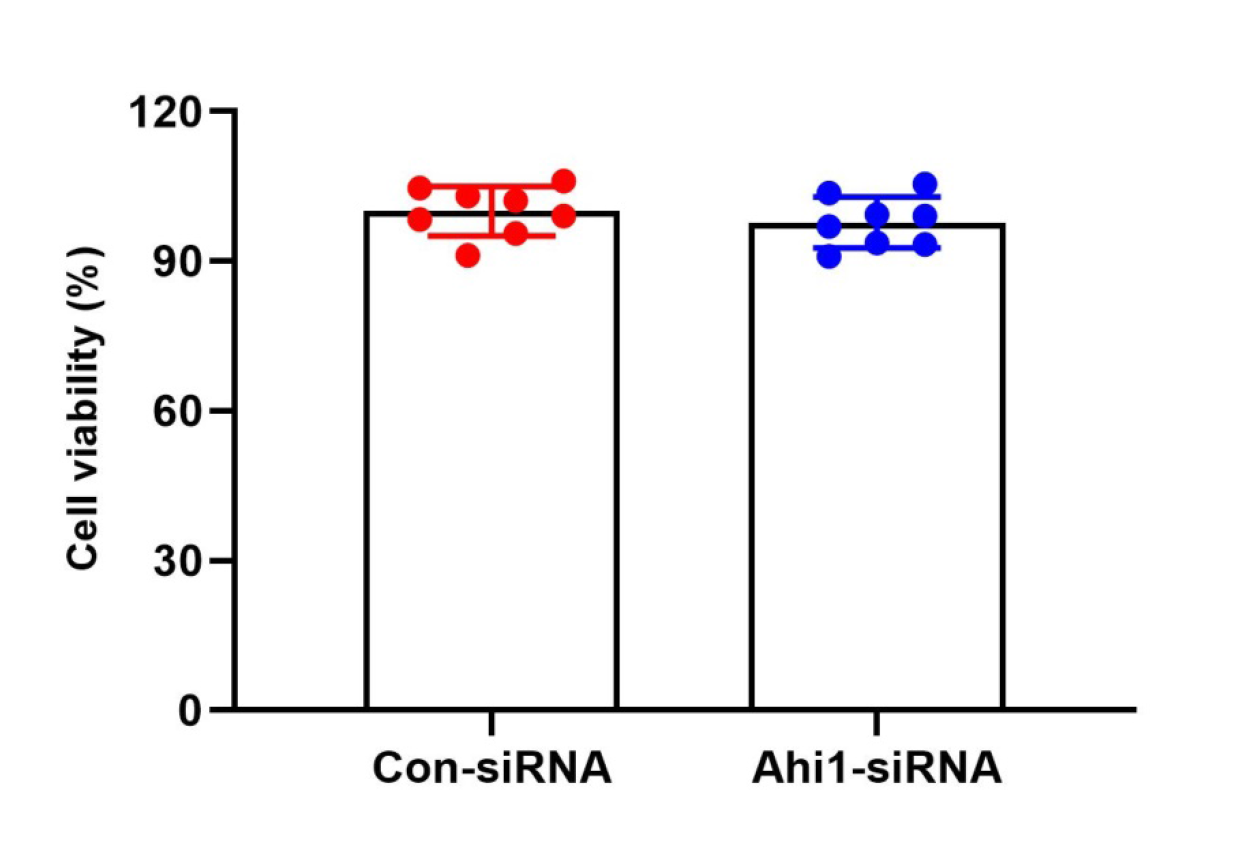

Supplement: Supplementary file 6 — Additional file 5: Fig. S4. Ahi1 knockdown did not alter cell viability. [file 12964_2022_1034_MOESM6_ESM.tif]

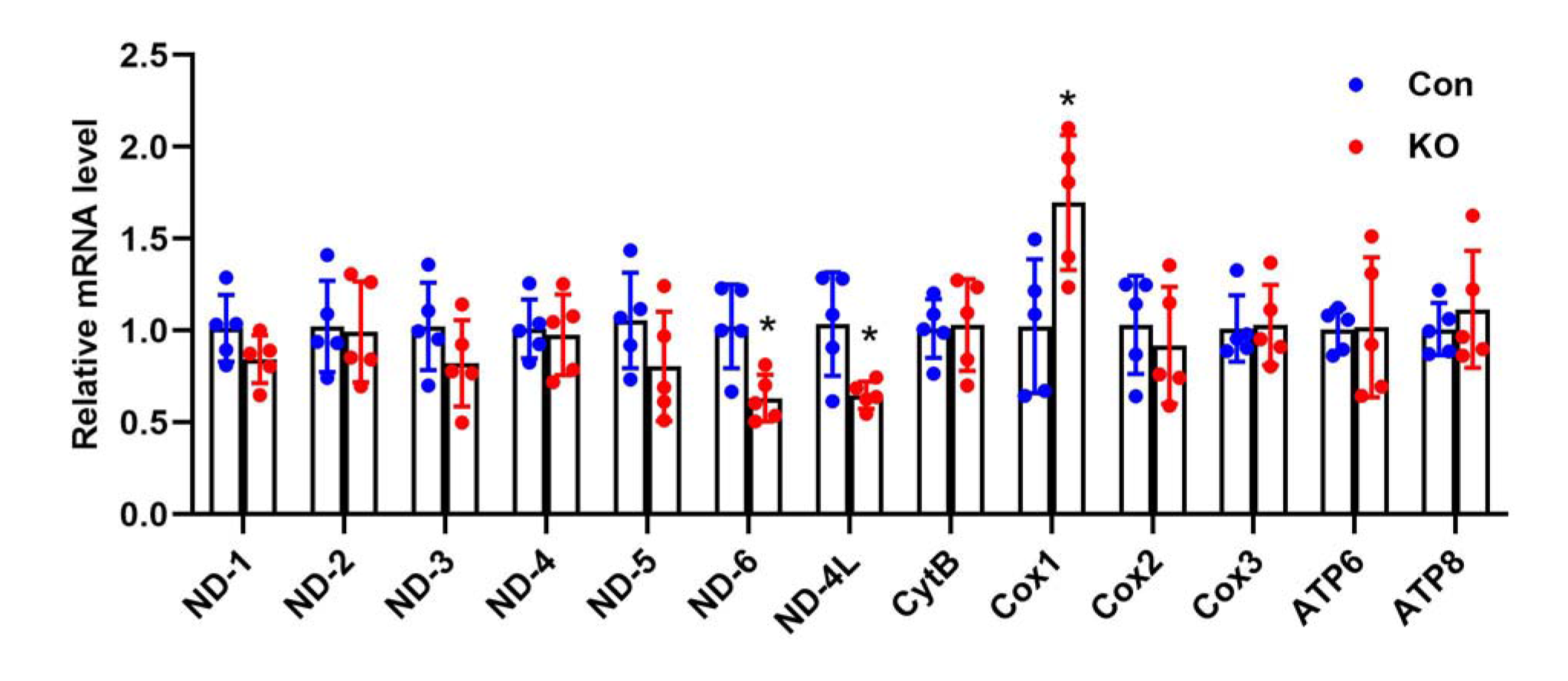

Supplement: Supplementary file 7 — Additional file 6: Fig. S5. Mitochondrial gene expression in the hypothalamus of Ahi1 KO mice was examined. [file 12964_2022_1034_MOESM7_ESM.tif]

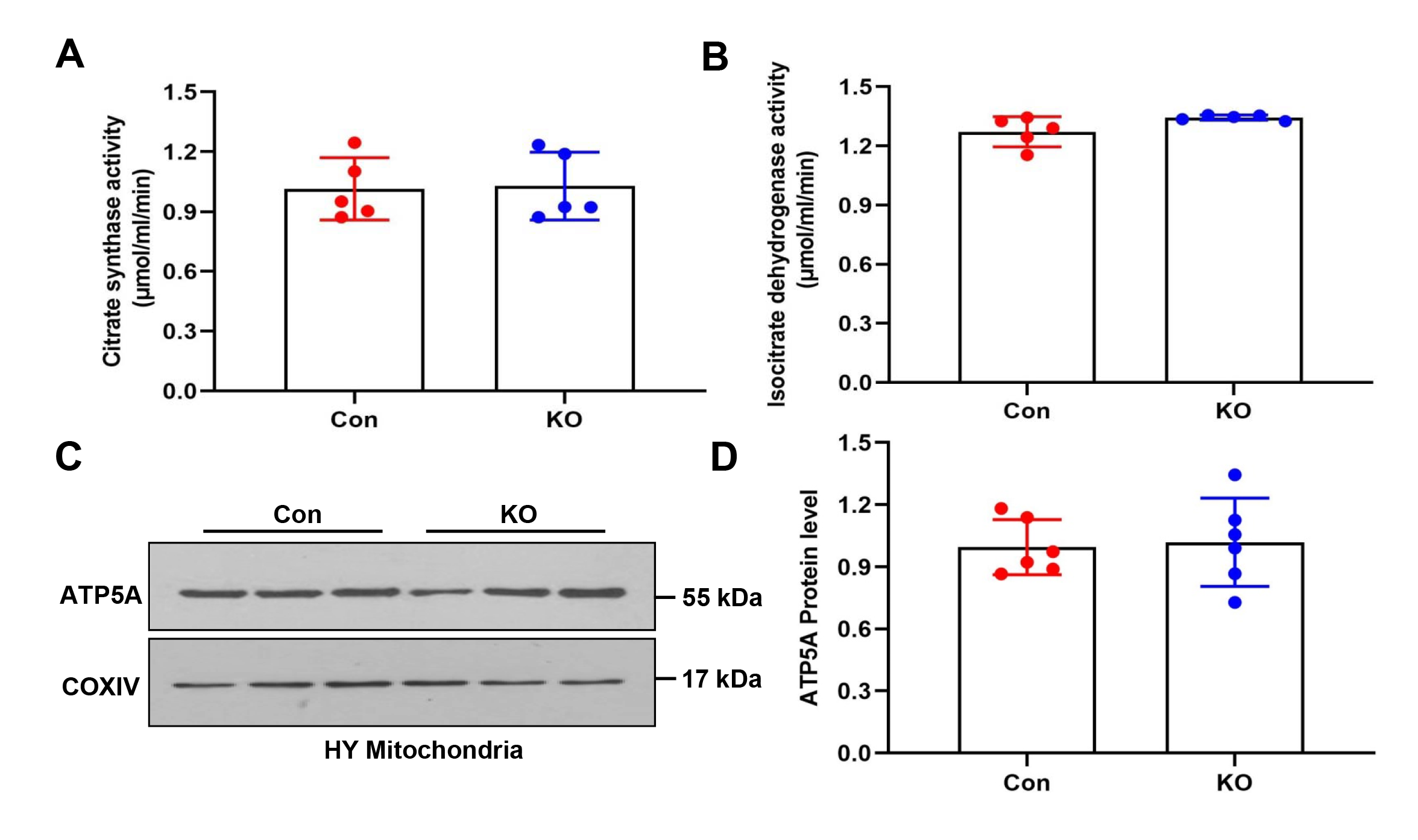

Supplement: Supplementary file 8 — Additional file 7: Fig. S6. Citrate synthase activity, isocitrate dehydrogenase activity, and ATP5A content were unchanged in Ahi1 KO mice. [file 12964_2022_1034_MOESM8_ESM.tif]

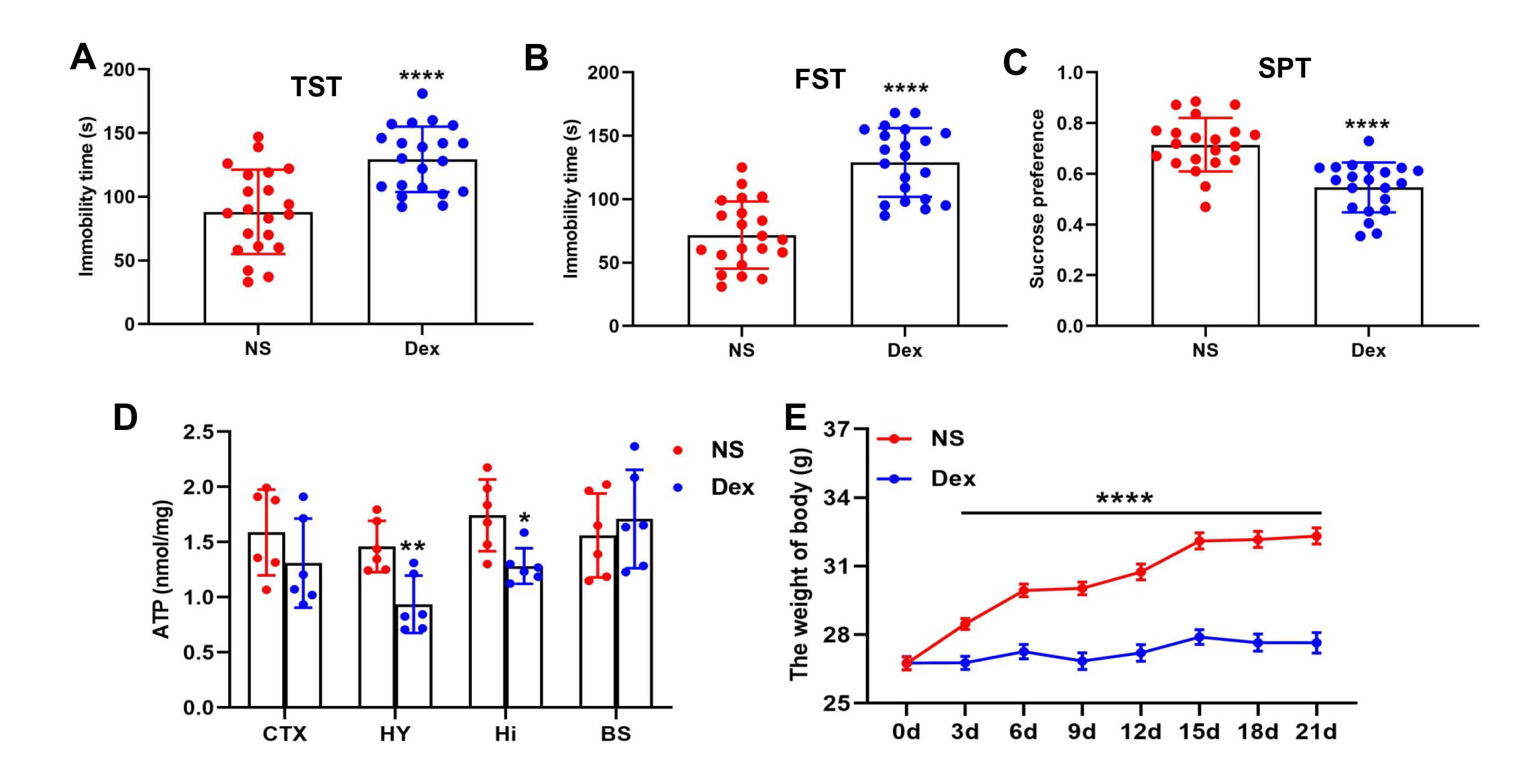

Supplement: Supplementary file 9 — Additional file 8: Fig. S7. Dexamethasone induced depression-like behavior in mice. [file 12964_2022_1034_MOESM9_ESM.tif]

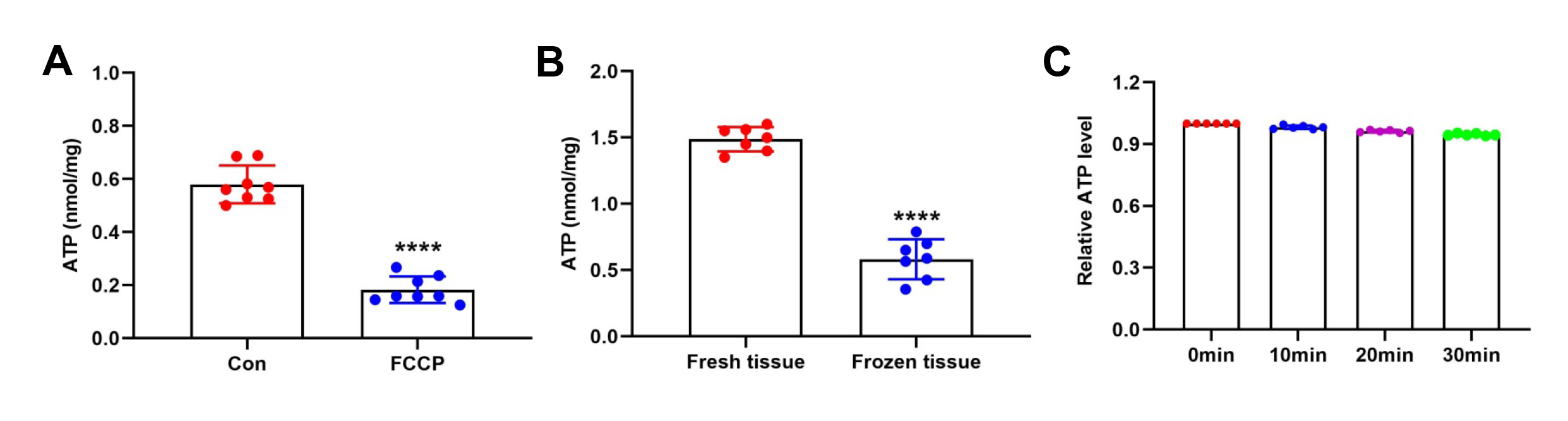

Supplement: Supplementary file 10 — Additional file 9: Fig. S8. ATP stability was evaluated by an ATP kit. [file 12964_2022_1034_MOESM10_ESM.tif]
